# Influence of Seeds’ Age and Clarification of Cold-Pressed Raspberry (*Rubus idaeus* L.) Oil on the DSC Oxidative Stability and Phase Transition Profiles

**DOI:** 10.3390/foods12020358

**Published:** 2023-01-12

**Authors:** Yolanda Victoria Rajagukguk, Mahbuba Islam, Jolanta Tomaszewska-Gras

**Affiliations:** Department of Food Quality and Safety Management, Poznań University of Life Sciences, ul. Wojska Polskiego 31/33, 60-637 Poznań, Poland

**Keywords:** raspberry seed oil, antioxidant, oxidative stability, DSC, seed residue

## Abstract

After cold-pressing, small particles of seed residue remain in raspberry seed oil (RSO), even after passing it through cold filtration. The removal of the remaining seed residue is rather an alternative option to improve the visual properties of RSO. This study investigated the influence that the seeds’ age (0, 10, 20 months) and clarification process after pressing has on the oxidative stability and phase transition of RSO by means of differential scanning calorimetry (DSC). The results proved that the oil centrifugation process reduces the DPPH radical scavenging activity and oxidative stability measured by p-anisidine value (p-AnV) and DSC oxidation induction time (OIT) at 120 °C of all RSO samples, regardless of the age of the seeds (*p* ≤ 0.05). No significant differences were observed on the DSC melting and crystallization properties at 1 °C/min after the oil clarification by centrifugation (*p* > 0.05). The storage time of raspberry seeds, i.e., 10 and 20 months after expiry date, influenced the quality deterioration of RSO, as measured by higher p-AnV, lower DPPH, and OIT values (*p* ≤ 0.05). The results presented provide new information about oil production processing, suggesting that producers should reconsider giving up the clarification process of oil, since it lowers all quality parameters.

## 1. Introduction

Raspberry seed oil (RSO) has received attention in recent years due to its promising health benefits [1,2]. RSO is often extracted using cold pressing because the resulting oil is safe for direct usage or even for consumption (i.e., cosmetics, salad dressing, cooking oil). Depending on the moisture content and heating parameters, the resulting oil contains certain proportions of solid particles (i.e., 0.0058–0.0608% *w*/*w*) [3], which are often called mechanical impurities [4]. Visible mechanical impurities that impact oil turbidity are separated by precipitation or cold filtration from the crude oil. The separation of mechanical impurities in RSO was reported to produce a minor reduction in oxidative stability [5]. As the presence of the remaining seed residue may affect visual properties, smaller seed particles in filtered oils can be completely removed by centrifugation, if this is necessary [6]. The necessity to completely remove the seed particles might lead to ambiguation for the RSO producer, as the raspberry seed itself contains significant amounts of natural antioxidants.

Additionally, it is important for the RSO producer to understand the characteristics of oil obtained from different seeds’ quality. Raspberry seeds are the by-product of raspberry fruit processing. Seeds obtained from by-product were mechanically damaged after fruit processing and needed to be processed further (i.e., sieving, drying) before oil extraction took place [7]. After the pre-treatment, the quality of raspberry seeds is influenced by the storage conditions (time, temperature) that may affect the hydrolytic and free-radical reactions in the seeds [8]. In view of the natural characteristics of seeds from by-product, RSO, and high consumer demand, an urgent need to develop better processing parameters to produce high-quality RSO has emerged.

The use of differential scanning calorimetry (DSC) as rapid oxidative stability and authenticity instruments for cold-pressed oils has been reported [9]. DSC allows to determine the thermal properties of oils based on their fatty acid and triacylglycerol compositions without any need to use hazardous chemicals. Despite its feasibility, there is limited knowledge on using DSC to measure compositional changes in seed oil. Several studies have reported quality changes in oils relating to raw material quality [10], cold filtration [5], and seed residue removal [11]. To the best of our knowledge, there is still little information regarding the influence that removing residue has on the oxidative stability and phase transition of RSO by DSC. Hence, the aim of this study is to assess (a) the influence of seed residue removal and (b) raw material quality on the oxidative stability and phase transition of RSO by DSC. The study was conducted to expand the application of DSC for oil quality and authenticity assessment.

## 2. Materials and Methods

**Figure 1:**
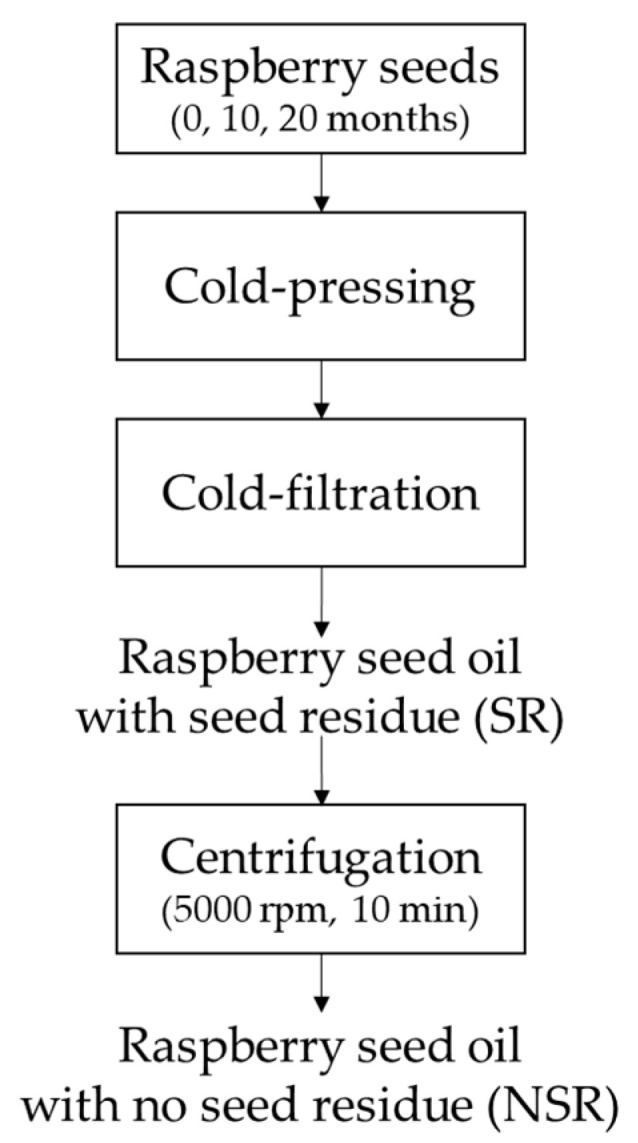
Flowchart to obtain raspberry seed oil (SR) and clarified without seed residue (NSR).

The samples consisted of six cold-pressed RSOs that differed in seed freshness and the presence of seed residue. Samples were cold-pressed from fresh seeds and expired seeds (10 and 20 months past their expiry dates, based on the label) as it is presented in Figure 1. Fresh and expired raspberry seeds were purchased from GreenField Sp. z o.o. (Warsaw, Poland). The seeds (300 g) were cold-pressed using oil presser (Counter Intelligence Oilpresso, The Netherlands) with a default outlet temperature at ±45 °C [12], cold filtered, and directly kept at the brown-glass bottle. All of the seeds were only went through a single pressing step. The acid value of the resulting oils were 6.59 ± 0.03, 10.06 ± 0.31, and 5.17 ± 0.00 mg KOH/g fat for oils from expired (20 and 10 months) and fresh seeds, respectively. The resulting oils were then divided into two groups: oils with seed residue (SR) and without seed residue (NSR). Oils without seed residue were obtained by centrifugation (MPW Med. Instruments, Warsaw, Poland) at 978× *g* (5000 rpm) for 10 min. The seed residue content in the centrifuged oils was approx. 5% (*w*/*w*). All oils were stored −80 °C before analysis with the headspace filled with nitrogen gas (99.99%) to minimize quality deterioration.

### 2.1. Chemical Analyses of Raspberry Seed Oil

#### 2.1.1. p-Anisidine Value

Analysis of p-anisidine value (p-AnV) was conducted following the method described in ISO 6885 [13]. The value represents the number of secondary oxidation products that react with the p-anisidine in acetic acid reagent. The absorbance reading was conducted at 350 nm.

#### 2.1.2. DPPH Radical Scavenging Activity

RSO (300 mg) were weighed and diluted with 0.3 mL of ethyl acetate. The resulting sample solution (100 µL) was mixed with DPPH radicals’ solution (1 mM, 250 µL) and 2 mL of ethyl acetate. After 20 min, the absorbance reading was conducted at 515 nm. DPPH value of RSO were expressed as µmol of Trolox equivalent (TE) per gram of oil.

### 2.2. Thermal Analysis of Raspberry Seed Oil

Thermal analysis by DSC was performed based on the method described in ISO 11357-6 [14]. The oxidative stability and melting-crystallization profile or raspberry seed oil were analyzed.

#### 2.2.1. Oxidative Stability Determination by Isothermal DSC

DSC isothermal analysis was performed at 120 and 140 °C, following the method reported by Islam et al. [15] DSC isothermal analysis provided information regarding several oxidative stability parameters, such as oxidation induction time (OIT), oxidation end time (OET), length of oxidation (∆t), heat flow (Y), and the oxidation rate. The samples were analyzed in two replications.

#### 2.2.2. Melting and Crystallization Profile by DSC

Melting and crystallization profiles were determined using the method described by Tomaszewska-Gras [16], with a slight modification. Melting curves were obtained by heating the sample from −70 to 30 °C at 1 °C/min. On the other hand, crystallization curves were obtained by cooling the sample from 30 to −70 °C at 1 °C/min. The measurement was conducted in two replications.

### 2.3. Statistical Analysis

Data analysis was conducted using Tibco Statistica 13.3 software (Tibco Software Inc., Tulsa, OK, USA). One-way ANOVA was carried out using Tukey’s multiple range test (α = 0.05).

## 3. Results and Discussion

### 3.1. Quality Characteristics of Cold-Pressed Raspberry Oil

Table 1 presents the quality characteristics of raspberry seed oils as measured by the p-anisidine value (p-AnV) and DPPH radical scavenging activity. The p-AnV expressed the amount of secondary oxidation product developed from peroxide decomposition. The analysis represented the advanced stage of oxidation that occurred in oils obtained from expired seeds. Oxidative stability measured by the p-AnV of unclarified oil with seed residue (SR) ranged from 4.56 to 11.53, whereas clarified with no-seed residue (NSR) oils ranged from 7.88 to 17.35. Generally, higher oxidative deterioration was observed in oils extracted from expired raspberry seeds. Significant differences in p-AnV among oils obtained from fresh (0 month) and expired raspberry seeds (10 and 20 months) were observed (*p* ≤ 0.05). These results were in agreement with another study that reported the increment of the p-AnV after retesting the oil a year later than the expiry dates [17]. Besides seed freshness, the oxidative deterioration of the oils was also influenced by the absence of seed residue. Raspberry seed oils from the NSR group exhibited a p-AnV up to 42% higher than the SR group (*p* ≤ 0.05). The results were in accordance with those of van Hoed et al. [5], who pointed out that filtering berry seed oil leads to a reduction in oil quality, as expressed by a higher p-AnV.

Furthermore, the findings on the p-AnV were supported by the results from DPPH analysis. The SR group exhibited higher DPPH radical scavenging activity (*p* ≤ 0.05), which resulted in a lower p-AnV than the NSR group. The results indicated the role of seed residue in protecting oil from oxidation. In contrast, another study reported that the presence of seed residue negatively impacted on the quality characteristics of cold-pressed sunflower oil [11]. It is worth noting that the influence of seed residue of oil depends on the natural characteristics of the seeds themselves. The ability of raspberry seed to protect lipid against oxidative damage was reported in another study [2]. The authors highlighted how the protective abilities of raspberry seed were influenced by the presence of bioactive component in seeds such as anthocyanin, namely cyanidin-3-o-rutinoside, as well as quercetin and other polyphenolic compounds. Additionally, raspberry seeds themselves were also considered a natural antioxidant in other studies, as they exhibited substantial amounts of ellagic acid, which was often linked to a significant antioxidative activity [1,18].

Seeds’ age is shown to influence the gradual reduction of DPPH scavenging activity in the NSR group (*p* ≤ 0.05). Older seeds might have been exposed to a longer peroxidation process that caused the depletion of natural antioxidant content in the resulting oil. Similar results regarding the reduction of DPPH value after storage of crude seed oil were reported by Chew et al. [19]. In contrast, no significant reduction in DPPH scavenging activity was observed between oils from expired seeds from the SR group (10 months and 20 months) (*p* > 0.05). Minor changes might be related to the protective ability from seed residue, as previously explained.

### 3.2. Influence of Seeds’ Age and Oil Clarification on Oxidative Stability

The oxidative stability of raspberry seed oil was investigated based on DSC isothermal parameters at 120 and 140 °C, as presented in Table 2. Oxidative induction time (OIT) at 120 °C of SR group ranged between 96.01–106.42 min, which was significantly higher than the NSR group that ranged between 86.22–90.65 min (*p* ≤ 0.05). The values of OIT in raspberry seed oil were in the same range as in previously reported studies [20]. In accordance with the p-AnV results (Table 1), oils with lower oxidative deterioration possessed a higher OIT value than oils extracted from older seeds. Significant differences in high OIT values were observed in 0-month oil from the SR group at 120 °C and in 0-month oils from both groups at 140 °C.

It is worth mentioning that despite the major differences in DPPH radical scavenging activity (Table 1) between the SR and NSR group, the OIT of the NSR group was only 10–16 min shorter. These results might indicate that DPPH radical scavenging activity does not significantly influence the OIT of oils. Oxidative stability in berry seed oil was linked to fatty acid composition, such as the content of polyunsaturated fatty acid and tocopherols [5]. Interestingly, differences in the OIT value between the SR and NSR groups were not observed at 140 °C (*p* > 0.05). The results indicated that the presence of seed residue does not have an impact on the oxidative stability of raspberry seed oil at higher temperatures i.e., 140 °C. It also indicates that a lower isothermal temperature, in this case 120 °C, is suitable for DSC oxidative stability assessment to differentiate oils that share similar general properties. These results show that the presence of substances with antioxidant activity from seed residue plays a significant role at the temperature of 120 °C, since the oxidation times (OIT) were very long, i.e., between 86.2 and 106.4 min., in contrast to the OIT test at 140 °C, where the time of oil resistance to oxidation was much shorter, between 22.8 and 27.7 min. Similar findings related to the ability of lower isothermal temperatures to differentiate oils from different seeds cultivar were reported in another study [15].

The length of oxidation in the SR group at 120 °C was shorter than in the NSR and ranged from 12.62–15.58 min (*p* ≤ 0.05). Oxidation in the NSR group occurred at a higher oxidation rate than in the SR group at 120 °C (*p* ≤ 0.05). The results indicate that raspberry seed oil from the NSR group became less stable than from the SR group, especially when the oil was exposed to a certain temperature (120 °C) for a longer period of OIT (86–90 min). In contrast, the oxidative stability observation at 140 °C showed that the NSR group had a lower oxidation rate when the OIT value was shorter (25–26 min). Following these results, seed freshness was shown to have an insignificant influence on the length of oxidation and the oxidation rate of oil at any of the isothermal temperatures observed (*p* > 0.05). Oxidative stability findings from the study might constitute an important approach for the seed oil industry to design the processing parameters of RSO intended for cooking, especially if it includes heat exposure.

### 3.3. Influence of Seed Freshness and Oil Clarification on Melting and Crystallization Profile

**Figure 2:**
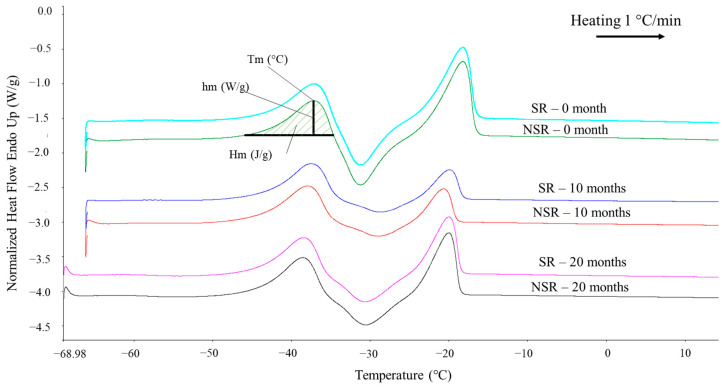
DSC melting curves of raspberry seed oil at scanning rate 1 °C/min. SR: oil not clarified, with seed residue, NSR: oil clarified, with no seed residue.

The DSC melting profiles of raspberry seed oil obtained at a scanning rate 1 °C/min are presented in Figure 2. Raspberry seed oil from both groups possessed three melting peaks that consist of two endothermic peaks (Tm1 ≅ −41 °C, Tm3 ≅ −22 °C) and one exothermic peak (Tm2 ≅ −39 °C). Similar DSC profiles of raspberry seed oil were previously reported in other studies [21,22].

The results of DSC melting and crystallization analysis of raspberry seed oil at a scanning rate 1 °C/min are presented in Table 3. The presence of seed residue was shown to influence slight differences in the DSC melting and crystallization parameters. However, these differences were very small. In contrast to the statistical results, no specific differences in the shape of melting peaks were observed between the SR and NSR group (Figure 2). Thermal behaviors were influenced by the fatty acid composition of the sample [23]. According to Parry & Liu [18], the fatty acid composition of raspberry seed and its oil were similar to each other. Raspberry seed and its oil were reported to contain high amounts of oleic (C18:1), linoleic (C18:2 *n*-6), and α-linolenic (C18:3 *n*-3) fatty acid. Additionally, the authors (Parry & Liu [18]) highlighted that major differences between raspberry seeds and its oil were found in the higher amount of polyunsaturated fatty acid (PUFA) in the oil. This might explain the minor differences in thermal properties between both groups, as presented in Table 3.

Concerning the influence of seeds’ age, the most distinctive differences were observed in the melting enthalpies (*ΔHm*) between oils from fresh (0 month) and older seeds (10 and 20 months) in both groups. The results showed that oils with better oxidative stability possessed higher enthalpies *ΔHm*1 and *ΔHm*3 than other oils from older seeds (*p* ≤ 0.05). A continuous oxidation process was reported to affect the gradual decrement of unsaturated fatty acid in blended raspberry seed oil [24]. The decreased amount of unsaturated fatty acid in oils from older seeds might linked to the lower melting enthalpies, as presented in Table 3.

## 4. Conclusions

The removal of seed residue significantly reduced the quality of RSO, as indicated by higher p-anisidine value, lower DPPH, and lower OIT 120 °C in the NSR group. No distinctive differences were observed in the DSC melting and crystallization profile of raspberry seed oil from SR and NSR groups. Additionally, seed freshness was reported to affect the quality characteristics, DSC oxidative stability, and melting properties of raspberry seed oil from the SR and NSR groups. To conclude, the intended use of the final RSO product should be considered before removing the seed residue. The clarification process might be beneficial to improve the visual appearance of RSO, but it lowers both the oxidative stability of the oil and its shelf life. This work provided new information about the processing parameters in raspberry seed oil production, which is of benefit to seed oil producers.

## Figures and Tables

**Table 1:** Chemical characteristics of raspberry seed oil based on p-anisidine value (p-AnV) and DPPH radical scavenging activity.

Analysis	Months after Exp. Dates	SR	NSR
p-AnV	20	11.53 ± 0.52 ^aB^	17.35 ± 0.09 ^bC^
	10	10.51 ± 0.12 ^aB^	13.27 ± 0.83 ^bB^
	0	4.56 ± 0.61 ^aA^	7.88 ± 0.12 ^bA^
DPPH (µmol TE/g Oil)	20	190.74 ± 1.72 ^bA^	18.96 ± 0.11 ^aA^
	10	189.53 ± 3.01 ^bA^	24.38 ± 0.81 ^aB^
	0	206.87 ± 2.21 ^bB^	30.88 ± 1.3 ^aC^

SR: oil with seed residue, NSR: oil with no seed residue. Values are given as mean ± SD (*n* = 3). Different letters (a, b) in the same row indicate a significant difference (*p* ≤ 0.05). Different letters (A, B, C) in the same column within the same analysis indicate a significant difference (*p* ≤ 0.05).

**Table 2:** Oxidative stability of raspberry seed oil based on DSC isothermal parameters (120 °C and 140 °C).

Isothermal (°C)	DSC Parameters	Months after Exp. Dates	SR	NSR
120	OIT (min)	20	96.51 ± 2.38 ^bA^	86.24 ± 1.94 ^aA^
		10	96.01 ± 2.44 ^bA^	86.22 ± 1.11 ^aA^
		0	106.42 ± 1.42 ^bB^	90.65 ± 3.70 ^aA^
	OET (min)	20	112.08 ± 1.22 ^aA^	113.02 ± 0.57 ^aA^
		10	108.63 ± 1.96 ^aA^	115.5 ± 2.58 ^aAB^
		0	122.01 ± 1.19 ^aB^	122.02 ± 1.15 ^aB^
	OIT-OET (min)	20	15.56 ± 1.16 ^aA^	29.78 ± 2.52 ^bA^
		10	12.62 ± 0.48 ^aA^	29.28 ± 1.47 ^bA^
		0	15.58 ± 2.61 ^aA^	31.37 ± 2.54 ^bA^
	Oxidation rate (W/g min)	20	0.003 ± 0.00 ^aA^	0.014 ± 0.00 ^bA^
		10	0.004 ± 0.00 ^aA^	0.017 ± 0.00 ^bA^
		0	0.003 ± 0.00 ^aA^	0.012 ± 0.00 ^bA^
140	OIT (min)	20	25.08 ± 0.8 ^aAB^	25.80 ± 0.73 ^aAB^
		10	23.43 ± 0.84 ^abA^	22.83 ± 1.14 ^aA^
		0	27.72 ± 0.90 ^aC^	26.87 ± 0.93 ^aC^
	OET (min)	20	35.56 ± 0.28 ^aAB^	37.86 ± 0.63 ^bA^
		10	33.24 ± 1.09 ^aA^	36.09 ± 0.15 ^aA^
		0	36.89 ± 0.51 ^aC^	40.78 ± 0.87 ^bB^
	OIT-OET (min)	20	10.48 ± 1.08 ^aA^	12.05 ± 1.36 ^aA^
		10	9.81 ± 0.25 ^aA^	13.26 ± 1.30 ^aA^
		0	9.18 ± 1.41 ^aA^	13.91 ± 1.81 ^aA^
	Oxidation rate (W/g min)	20	0.067 ± 0.00 ^aA^	0.049 ± 0.01 ^aA^
		10	0.073 ± 0.01 ^bA^	0.043 ± 0.00 ^aA^
		0	0.081 ± 0.01 ^bA^	0.042 ± 0.00 ^aA^

SR: oil not clarified, with seed residue, NSR: oil clarified, with no seed residue. Values are given as mean ± SD (*n* = 2). Different letters (a, b) in the same row indicate a significant difference (*p* ≤ 0.05). Different letters (A, B, C) in the same column within the same analysis and the same DSC parameters indicate a significant difference (*p* ≤ 0.05). OIT: oxidation induction time, OET: oxidation end time.

**Table 3:** DSC melting and crystallization profile of raspberry seed oil at scanning rate 1 °C/min.

Melting		Months after Exp. Dates	SR	NSR
Peak temperature (°C)	Tm1	20	−41.02 ± 0.01 ^aB^	−40.98 ± 0.23 ^aA^
		10	−41.16 ± 0.19 ^aAB^	−41.41 ± 0.04 ^aA^
		0	−41.60 ± 0.01 ^aA^	−41.42 ± 0.02 ^bA^
	Tm2	20	−38.79 ± 0.04 ^aA^	−39.07 ± 0.23 ^aA^
		10	−38.92 ± 0.23 ^aA^	−39.34 ± 0.05 ^aA^
		0	−39.19 ± 0.01 ^aA^	−39.08 ± 0.00 ^bA^
	Tm3	20	−21.50 ± 0.08 ^bA^	−21.94 ± 0.04 ^aA^
		10	−21.76 ± 0.33 ^aA^	−22.00 ± 0.01 ^aA^
		0	−24.22 ± 4.89 ^aA^	−21.77 ± 1.63 ^aA^
Peak height (W/g)	hm1	20	0.07 ± 0.000 ^aA^	0.08 ± 0.001 ^bA^
		10	0.09 ± 0.003 ^aB^	0.09 ± 0.000 ^aB^
		0	0.07 ± 0.003 ^aA^	0.08 ± 0.004 ^aA^
	hm2	20	−0.26 ± 0.006 ^bA^	−0.34 ± 0.004 ^aA^
		10	−0.27 ± 0.009 ^aA^	−0.30 ± 0.023 ^aA^
		0	−0.20 ± 0.004 ^aB^	−0.22 ± 0.020 ^aB^
	hm3	20	0.27 ± 0.00 ^aB^	0.29 ± 0.01 ^bB^
		10	0.25 ± 0.00 ^aA^	0.26 ± 0.01 ^aA^
		0	0.31 ± 0.00 ^aC^	0.32 ± 0.00 ^bC^
Enthalpy (J/g)	*ΔHm*1	20	20.81 ± 1.73 ^aA^	25.30 ± 2.18 ^aA^
		10	38.32 ± 6.43 ^aAB^	36.73 ± 1.51 ^aAB^
		0	44.23 ± 3.11 ^aB^	45.26 ± 4.84 ^aB^
	*ΔHm*2	20	−23.96 ± 1.46 ^aA^	−26.34 ± 0.32 ^aA^
		10	−24.01 ± 1.21 ^aA^	−24.15 ± 0.66 ^aA^
		0	−16.13 ± 0.45 ^aB^	−16.52 ± 1.56 ^aB^
	*ΔHm*3	20	65.48 ± 0.11 ^aA^	72.71 ± 0.59 ^bA^
		10	76.02 ± 2.02 ^aB^	80.23 ± 1.74 ^aA^
		0	90.55 ± 0.33 ^aC^	93.65 ± 3.21 ^aB^
Crystallization		Months after exp. dates	SR	NSR
Peak temperature (°C)		20	−63.72 ± 0.06 ^aA^	−62.15 ± 1.51 ^aA^
		10	−63.57 ± 0.4 ^aA^	−63.67 ± 0.26 ^aA^
		0	−60.64 ± 0.01 ^aB^	−60.71 ± 0.16 ^aA^
Peak height (W/g)		20	−0.08 ± 0.003 ^bC^	−0.10 ± 0.002 ^aB^
		10	−0.09 ± 0.001 ^bB^	−0.11 ± 0.003 ^aB^
		0	−0.13 ± 0.001 ^bA^	−0.14 ± 0.002 ^aA^
Enthalpy (J/g)		20	−26.07 ± 1.79 ^aA^	−28.29 ± 2.46 ^aA^
		10	−28.6 ± 1.32 ^aA^	−28.91 ± 0.56 ^aA^
		0	−28.99 ± 1.34 ^aA^	−31.21 ± 0.04 ^aA^

SR: oil not clarified, with seed residue, NSR: oil clarified, with no seed residue. Values are given as mean ± SD (*n* = 2). Different letters (a, b) in the same row indicate a significant difference (*p* ≤ 0.05). Different letters (A, B, C) in the same column within the same analysis and the same DSC parameters indicate a significant difference (*p* ≤ 0.05).

## Data Availability

The data used to support the findings of this study can be made available by the corresponding author upon request.
